# Prevalence, virulence potential, and pulsed-field gel electrophoresis profiling of Shiga toxin-producing *Escherichia coli* strains from cattle

**DOI:** 10.1186/s13099-017-0169-x

**Published:** 2017-04-21

**Authors:** Hee-Jin Dong, Soomin Lee, Woohyun Kim, Jae-Uk An, Junhyung Kim, Danil Kim, Seongbeom Cho

**Affiliations:** 1 BK21 PLUS Program for Creative Veterinary Science Research, College of Veterinary Medicine and Research Institute for Veterinary Science, Seoul National University, Seoul, 08826 South Korea; 2 Department of Farm Animal Medicine, Seoul National University, Seoul, 08826 Republic of Korea; 3 Farm Animal Clinical Training and Research Center, Institute of Green-Bio Science and Technology, Seoul National University, Pyeongchang, 25354 Republic of Korea

**Keywords:** STEC, Cattle, *stx* variant, Antimicrobial resistance, Virulence gene, PFGE

## Abstract

**Background:**

As a primary source of Shiga-toxin-producing *Escherichia coli* (STEC) infection, cattle are often targeted to develop strategies for reducing STEC contamination. Monitoring the virulence potentials of STEC isolates from cattle is important for tracing contamination sources, managing outbreaks or sporadic cases, and reducing the risks for human infection. This study aimed to investigate the prevalence of STEC in cattle farm samples in South Korea and to assess their virulence potentials.

**Results:**

In total, 63 STEC were isolated from 496 cattle farm samples, and temperature and rainfall affected STEC prevalence (*p* < 0.001). The O157 serogroup was most prevalent, followed by O108, O8, O84, O15, and O119. In the *stx* variant test, high prevalence of *stx2a* and *stx2c* (known to be associated with high STEC virulence) were observed, and *stx2g*, a bovine STEC variant, was detected in STEC O15 and O109. Additionally, *stx1c* was detected in *eae*-positive STEC, suggesting genetic dynamics among the virulence genes in the STEC isolates. STEC non-O157 strains were resistant to tetracycline (17.9%), ampicillin (14.3%), and cefotaxime (3.6%), while STEC O157 was susceptible to all tested antimicrobials, except cefotaxime. The antimicrobial resistance genes, *bla*_*TEM*_ (17.5%), *tetB* (6.3%), and *tetC* (4.8%), were only detected in STEC non-O157, whereas *tetE* (54.0%) was detected in STEC O157. *AmpC* was detected in all STEC isolates. Clustering was performed based on the virulence gene profiles, which grouped STEC O84, O108, O111, and O157 together as potentially pathogenic STEC strains. Finally, PFGE suggested the presence of a prototype STEC that continues to evolve by genetic mutation and causes within- and between-farm transmission within the Gyeonggi province.

**Conclusions:**

Considerable numbers of STEC non-O157 were isolated from cattle farms, and the virulence and antimicrobial resistance features were different between the STEC O157 and non-O157 strains. STEC from cattle with virulence or antimicrobial resistance genes might represent a threat to public health and therefore, continual surveillance of both STEC O157 and non-O157 would be beneficial for controlling and preventing STEC-related illness.

## Background

Since the identification of STEC O157:H7 as a foodborne zoonotic disease in 1982 [1], human infections by STEC have been reported worldwide [2, 3]. While numerous studies have focused on STEC O157:H7, the most well-known and notorious serotype, >400 serotypes of STEC non-O157 have been implicated as etiological agents of several outbreaks and in sporadic cases of STEC infection [4]. Recently, STEC non-O157 infection cases have increased globally, highlighting the significance of investigating STEC non-O157 [3, 5, 6]. Among the STEC non-O157 serotypes, O26, O45, O103, O111, O121, and O145, were reported as the six major STEC non-O157 linked to human diseases [7, 8]. Scallen et al. reported that ~63,000 and 112,000 cases of foodborne illness caused by STEC O157 and non-O157, respectively, occur in the United States annually [2]. The progression of STEC infection varies, causing symptoms ranging from mild gastrointestinal symptoms to severe hemorrhagic colitis (HC) or hemolytic uremic syndrome (HUS) [9–11]. Predicting the risk of STEC is especially important for public health because STEC infection might develop into a life-threatening disease, and is often associated with large and multinational outbreaks [10, 12, 13].

Although the pathogenicity of STEC is not fully understood, several virulence factors have been identified [10], including Shiga toxins, intimin, and the 60-MDa plasmids (enterohemolysin or serine protease) [10]. Shiga toxins are the principal virulence factors of STEC, and two major types of Shiga toxins are known, Stx1 and 2 [14]. While the DNA sequence of *stx1* is highly conserved and only a few *stx1* variants have been reported (including *stx1c* and *stx1d*), the *stx2* sequence shows 84–99% similarity among the *stx* *2* variants [10, 15]. Because the variants are related to the properties of Shiga toxin, subtyping of the *stx* variants is important for predicting the virulence potential of STEC in human infection [16]. Among Shiga toxin and its variants, Stx2 is most associated with severe disease [17, 18]. Stx2 is a 1000 times more toxic than Stx1 to renal microvascular endothelial cells, and Stx2 and Stx2c are more commonly reported in HUS patients [19–21]. Intimin, one of the proteins encoded by *eae* in the locus of enterocyte effacement, which is responsible for the formation of attaching and effacing (A/E) legions [10, 22, 23]. Several other factors also contribute to the virulence of STEC. EhxA (EHEC-enterohemolysin) disrupts the cytoplasmic membranes of mammalian cells [10, 24]. EspP (a serine protease) potentiates STEC colonization in the human gut [25, 26], and KatP (catalase peroxidase) [27], SubAB (subtilase), and Saa (STEC autoagglutinating adhesin) are associated with the virulence of STEC [14, 28, 29].

Cattle are a primary source of STEC infection and are often targeted to develop strategies for reducing contamination. Therefore, monitoring the virulence potentials of STEC isolates from cattle is important for tracing the sources of contamination, managing outbreaks or sporadic cases, and reducing the risks for human infection. This study investigated the prevalence of STEC O157 and non-O157 in cattle farm samples in South Korea and assessed the virulence potentials of STEC isolates from these samples by characterizing *stx* variants, antimicrobial resistance, and virulence genes. Finally, genetic analysis was performed to analyze the genetic dynamics of STEC strains isolated over a 4-year period.

## Methods

### Sample collection

Samples were collected from 15 cattle farms located in the Gyeonggi province in Korea during 2012–2015. Each farm was visited one to nine times during the sampling period (median = 1, average = 1.9), and cattle farm samples, including feces, ground soil, and water, were collected. Fecal samples were collected by direct rectal retrieval using disposable gloves. Environmental samples in the farm were collected using sterilized spatulas. Each sample collected had a mass of at least 5 g (or a volume of at least 5 mL for liquid samples). A total of 469 samples (419 fecal, 47 ground soil, one water, one raw milk, and one forage sample) were collected and transported immediately to the laboratory for STEC isolation.

### Isolation of STEC strains

Shiga-toxin-producing *Escherichia coli* O157 was isolated using a combination of standard culture methods and immunomagnetic separation methods, as described previously [30]. Briefly, 1 g or 1 mL of each sample was homogenized for 1 min with 9 mL of modified EC broth (Becton, Dickinson and Company, USA) supplemented with novobiocin (20 mg/L, Oxoid, USA) using a homogenizer, and then incubated overnight at 37 °C. The enriched culture suspension was then mixed with anti-*E. coli* O157 antibody-coated magnetic beads (Dynal, Invitrogen, USA) and separated using a magnetic rack, as described in the manufacturer’s guidelines. The suspension was then tested for the formation of typical colonies in tellurite-added sorbitol MacConkey agar (T-SMAC; BD, USA), MacConkey agar (MAC; BD, USA), and CHROMagar O157 (CHROMagar Microbiology, France). To confirm STEC O157 isolation, the serotype of the colonies was tested using the *E. coli* O157 Latex Test Kit (Oxoid, UK).

To isolate STEC non-O157, multiplex polymerase chain reaction (PCR) combined with standard culture methods was used, as described previously [30]. Briefly, one loop of enriched culture was streaked onto T-SMAC and tested for the presence of Shiga toxin genes by PCR as described below in the virulence gene profiling section. The serotype of colonies harboring *stx* genes was then tested by conventional agglutination tests, using *E. coli* antisera (JoongKyeom, Ansan, Korea).

### Antimicrobial-susceptibility test

A standard disk-diffusion test was performed to determine antimicrobial susceptibility for the following 14 antimicrobial drugs: ampicillin (AM, 10 µg), chloramphenicol (C, 30 µg), imipenem (IMP, 10 µg), tetracycline (TE, 30 µg), amikacin (AN, 30 µg), amoxicillin–clavulanic acid (AMC, 20/10 µg), ceftazidime (CAZ, 30 µg), gentamicin (GM, 10 µg), nalidixic acid (NA, 30 µg), trimethoprim–sulfamethoxazole (STX, 1.25/23.75 µg), ceftriaxone (CRO, 30 µg), aztreonam (ATM, 30 µg), cefotaxime (CTX, 30 µg), and cefpodoxime (CPD, 10 µg). For quality control, *E. coli* ATCC 25922 was used as the reference strain. Antimicrobial susceptibility was interpreted as guided by the Clinical Laboratory Standard Institute (CLSI).

ESBL-producing STEC was detected following the CLSI ESBL phenotypic confirmatory test, which is a disk-diffusion test. Briefly, STEC isolates found to be resistant to cefpodoxime, ceftazidime, aztreonam, cefotaxime, or ceftriaxone were screened in the phenotypic confirmatory test. A standard double-disk test was performed to confirm the ESBL phenotype, as described in the NCCL guidelines. Briefly, enriched STEC isolates were adjusted to 0.5 McFarland scale (McF), and 100 µL of the suspension was spread onto Mueller–Hinton agar. Antimicrobial disks containing ceftazidime + ceftazidime–clavulanic acid or cefotaxime + cefotaxime–clavulanic acid were placed on STEC-inoculated MH agar and incubated at 35 °C for 18 h. The ESBL phenotype was considered positive when the zone diameter resulting from the combination of the antimicrobial agent and clavulanic acid was > 5 mm larger than that obtained using the antimicrobial test agent alone.

### Detection of stx variants, virulence genes, and antimicrobial resistance genes

The presence of virulence and antimicrobial resistance genes was determined by PCR using a MyCycler thermal cycler (Bio-Rad Laboratories, USA). For DNA preparation, a single colony of each isolate was suspended in 1 mL of normal saline and centrifuged for 3 min at 6000×*g*. The pellets were re-suspended with 200 µL of sterile water and boiled for 10 min. The suspension was centrifuged for 3 min at 6000×*g*, and the supernatant was used as the DNA template. PCR was conducted as described previously to detect Shiga toxin genes (*stx1, stx1c, stx1d, stx2, stx2a, stx2c, stx2d, stx2e, stx2f,* and *stx2* *g*), virulence genes (*eae*, *tir*, *espB*, *espD, ehxA, katP, espP, iha, subA, stcE,* and *saa*), and antimicrobial resistance genes (*ampC, tetA, tetB, tetC, tetD, tetE, tetG, cat, cml, bla*_*OXA*_ *, bla*_*CMY*_ *, bla*_*TEM*_, and *qnr*). The primer sequences and reaction conditions for each gene are summarized in Table 1.

**Table 1:** Primer sequences and the PCR conditions used in this study

Target gene	Nucleotide sequences (5′–3′)	PCR conditions				Amplicon size (bp)	References
		Denaturation	Annealing	Extension	Cycle		
*stx* *1*	CAGTTAATGTGGTGGCGAAGG	94 °C, 60 s	56 °C, 60 s	72 °C, 60 s	25	348	[58]
	CACCAGACAATGTAACCGCTG						
*stx* *2*	ATCCTATTCCCGGGAGTTTACG	94 °C, 60 s	56 °C, 60 s	72 °C, 60 s	25	584	[58]
	GCGTCATCGTATACACAGGAGC						
*stx* *1c*	TTTTCACATGTTACCTTTCCT	94 °C, 60 s	56 °C, 60 s	72 °C, 60 s	30	498	[42]
	CATAGAAGGAAACTCATTAGG						
*stx* *1d*	CTTTTCAGTTAATGCGATTGCT	94 °C, 60 s	56 °C, 60 s	72 °C, 60 s	30	192	[42]
	AACCCCATGATATCGACTGC						
*stx* *2a*	GCGATACTGRGBACTGTGGCC	94 °C, 50 s	65 °C, 40 s	72 °C, 30 s	25	349	[59]
	CCGKCAACCTTCACTGTAAATGTG						
*stx* *2c*	GCGGTTTTATTTGCATTAGT	94 °C, 60 s	56 °C, 60 s	72 °C, 60 s	30	124	[42]
	AGTACTCTTTTCCGGCCACT						
*stx* *2d*	GGTAAAATTGAGTTCTCTAAGTAT	94 °C, 60 s	56 °C, 60 s	72 °C, 60 s	30	175	[42]
	CAGCAAATCCTGAACCTGACG						
*stx* *2e*	ATGAAGAAGATGTTTATAGCG	94 °C, 60 s	56 °C, 60 s	72 °C, 60 s	30	267	[42]
	TCAGTTAAACTTCACCTGGGC						
*stx* *2f*	AGATTGGGCGTCATTCACTGGTTG	94 °C, 60 s	56 °C, 60 s	72 °C, 60 s	30	428	[42]
	TACTTTAATGGCCGCCCTGTCTCC						
*stx* *2g*	GTTATATTTCTGTGGATATC	94 °C, 60 s	56 °C, 60 s	72 °C, 60 s	30	573	[42]
	GAATAACCGCTACAGTA						
*eae*	ATTACTGAGATTAAGGCTGAT	94 °C, 20 s	58 °C, 20 s	72 °C, 90 s	35	682	[4]
	ATTTATTTGCAGCCCCCCAT						
*tir*	CATTACCTTCACAAACCGAC	94 °C, 40 s	57 °C, 60 s	72 °C, 75 s	30	1550	[60]
	CCCCGTTAATCCTCCCAT						
*esp* *B*	GCCGTTTTTGAGAGCCAGAAT	94 °C, 40 s	63 °C, 45 s	72 °C, 60 s	30	633	[60]
	ATCATCCTGCGCTCTGCGAAC						
*etp* *D*	CGTCAGGAGGATGTTCAG	94 °C, 30 s	54 °C, 60 s	72 °C, 90 s	30	1062	[61]
	CGACTGCACCTGTTCCTGATTA						
*ehx* *A*	GTTTATTCTGGGGCAGGCTC	94 °C, 60 s	56 °C, 60 s	72 °C, 60 s	25	166	[58]
	CTTCACGTCACCATACATAT						
*Kat* *P*	CTTCCTGTTCTGATTCTTCTGG	94 °C, 30 s	58 °C, 60 s	72 °C, 150 s	30	2125	[27]
	AACTTATTTCTCGCATCATCC						
*esp* *P*	AAACAGCAGGCACTTGAACG	94 °C, 30 s	58 °C, 60 s	72 °C, 150 s	30	1830	[25]
	GGAGTCGTCAGTCAGTAGAT						
*iha*	CTGGCGGAGGCTCTGAGATCA	94 °C, 60 s	57 °C, 60 s	72 °C, 120 s	30	827	[62]
	TCCTTAAGCTCCCGCGGCTGA						
*sub* *A*	CGGCTTATCATCCTGTCAGC	94 °C, 45 s	57 °C, 60 s	74 °C, 60 s	30	233	[63]
	TATAGCTGTTGCTTCTGACG						
*stc* *E*	GGCTCCGGAGGTGGGGGAAT	94 °C, 30 s	60 °C, 60 s	72 °C, 15 s	30	399	[53]
	GAAGCCGGTGGAGGAACGGC						
*saa*	CGTGATGAACAGGCTATTGC	94 °C, 60 s	56 °C, 60 s	72 °C, 60 s	30	119	[56]
	ATGGACATGCCTGTGGCAAC						
*tetA*	GCTACATCCTGCTTGCCTTC	95 °C, 60 s	58 °C, 60 s	72 °C, 60 s	30	210	[64]
	CATAGATCGCCGTGAAGAG						
*tetB*	TTGGTTAGGGGCAAGTTTTG	95 °C, 60 s	56 °C, 60 s	72 °C, 60 s	30	659	[64]
	GTAATGGGCCAATAACACCG						
*tetC*	CTTGAGAGCCTTCAACCCAG	95 °C, 60 s	58 °C, 60 s	72 °C, 60 s	30	418	[64]
	ATGGTCGTCATCTACCTGCC						
*tetD*	AAACCATTACGGCATTCTGC	95 °C, 60 s	60 °C, 60 s	72 °C, 60 s	30	787	[64]
	GACCGGATACACCATCCATC						
*tetE*	AAACCACATCCTCCATACGC	95 °C, 60 s	58 °C, 60 s	72 °C, 60 s	30	278	[64]
	AAATAGGCCACAACCGTCAG						
*tetG*	GCTCGGTGGTATCTCTGCTC	95 °C, 60 s	60 °C, 60 s	72 °C, 60 s	30	468	[64]
	AGCAACAGAATCGGGAACAC						
*ampC*	CCCCGCTTATAGAGCAACAA	94 °C, 60 s	61 °C, 120 s	72 °C, 180 s	35	634	[65]
	TCAATGGTCGACTTCACACC						
*catA1*	AGTTGCTCAATGTACCTATAACC	95 °C, 60 s	57 °C, 70 s	72 °C, 120 s	32	547	[64]
	TTGTAATTCATTAAGCATTCTGCC						
*cmlA*	CCGCCACGGTGTTGTTGTTATC	95 °C, 60 s	57 °C, 70 s	72 °C, 120 s	32	698	[64]
	CACCTTGCCTGCCCATCATTAG						
*qnr*	TATCTCCCTGTCGTTCCAG	94 °C, 30 s	52 °C, 30 s	72 °C, 30 s	30	399	[66]
	AGAACTCGCCGATCAATG						
*bla*_*CMY*_	TGGCCAGAACTGACAGGCAAA	94 °C, 60 s	49 °C, 90 s	72 °C, 60 s	35	462	[67]
	TTTCTCCTGAACGTGGCTGGC						
*bla*_*OXA*_	TATCTACAGCAGCGCCAGTG	94 °C, 60 s	62 °C, 120 s	72 °C, 60 s	31	199	[65]
	CGCATCAAATGCCATAAGTG						
*bla*_*TEM*_	TACGATACGGGAGGGCTTAC	94 °C, 60 s	62 °C, 60 s	72 °C, 60 s	30	717	[68]
	TTCCTGTTTTTGCTCACCCA						

### Virulence gene profiling

A phylogenetic dendrogram of the virulence profiles was constructed by using the unweighted pair group method with arithmetic mean analysis (UPGMA) for binary data using BioNumerics, version 6.6 (Applied Maths NV, Belgium).

### Pulsed-field gel electrophoresis (PFGE)

Pulsed-field gel electrophoresis was performed following the CDC PulseNet protocol using CHEF MAPPER (Bio-Rad, Hercules, CA, USA). Briefly, STEC colonies were suspended in cell suspension buffer (100 mM Tris: 100 mM EDTA, pH 8.0) and then adjusted to the 4.0 McFarland scale (McF). The adjusted cell suspension (400 µL) was mixed gently with 20 µL of proteinase K (20 mg/mL) and 400 µL of 1% SeaKem Gold melted agarose gel to build a plug. The plug was soaked in a proteinase K-containing cell lysis buffer (50 mM Tris: 50 mM EDTA, pH 8.0 + 1% sarcosyl) for 2 h to lyse the cells, after which it was washed twice with sterile water for 15 min and then four times with TE buffer (10 mM Tris: 1 mM EDTA, pH 8.0) for 15 min. The plug was then digested with 50 U of *Xba*I restriction enzyme for 2 h. PFGE was performed with a pulse time of 2.16–54.17 s for STEC O157 and 6.76–35.38 s for STEC non-O157; *S.* Braenderup ATCC BAA664 was used as a size ladder marker. A Dice similarity coefficient with a UPGMA dendrogram was generated based on 1.5% tolerance windows and 1.5% optimization, using BioNumerics, version 6.6 (Applied Maths NV, Belgium).

### Statistical analysis

To identify factors potentially associated with prevalence, farm-management factors, environmental factors, and animal information were collected, if present (Table 2). Farm-management factors (farm size, ground soil hygiene, and diet), and animal information (age and breed) were obtained from the veterinarian in charge of each farm. Environmental factors included average temperature on sampling date, humidity, and rainfall within 3 days prior to sampling, which were obtained from the data provided by the Meteorological Administration (http://www.kma.go.kr/weather/observation/past_table.jsp). The association between STEC prevalence and farm management and environmental factors was analyzed by using the Chi squared test, and the association between STEC prevalence and animal factors was analyzed by Fisher’s exact test. The statistical analysis was performed using SPSS, version 22.0 (SPSS IBM, New York, NY, USA), and the variables were considered to be significantly associated when the *p* value was <0.05.

**Table 2:** Data summary of 15 cattle farms and their STEC prevalence

Farm	Farm size	Hygiene of sawdust^a^	Diet	Location	No of visit	Temperature^c^ (°C)	Humidity	Rain^b,c^	Sampling date (YYYYMM)	No. of samples collected	No. of STEC isolates	Farm prevalence	ID (breed, serotype)^e^ of STEC isolates
1	250	High	TMR^d^	Gapyeong	1	22.5	93.4	Yes	201208	8	0	0.00	
2	60	Mid	TMR	Gapyeong	1	22.5	93.4	Yes	201208	10	0	0.00	
3	60	Low	TMR	Gapyeong	1	22.5	93.4	Yes	201208	8	0	0.00	
4	50	Low	Hay	Cheongpyeong	2	22.4	80.1	Yes	201208	9	4	44.44	0823-2 (D, O157), 0823-4 (D, O157), 0823-5 (D, O157), 0823-8 (D, O157)
						21.4	78.6	Yes	201309	6	1	16.67	0909-5 (D, O157)
5	30	Low	TMR	Gapyeong	1	22.4	80.1	Yes	201208	7	0	0.00	
6	40	High	TMR	Gapyeong	1	23.1	80.9	Yes	201208	9	0	0.00	
7	160	High	TMR	Gapyeong	2	27.7	69	Yes	201208	10	9	90.00	0827-1 (D, O157), 0827-2 (D, O157), 0827-3 (D, O157), 0827-5 (D, O157), 0827-6 (D, O157), 0827-7 (D, O157), 0827-8 (D, O157), 0827-9 (D, O157), 0827-10 (G, O157)
						20	98.9	Yes	201309	8	1	12.50	0911-3 (D, NT)
8	150	High	TMR	Gapyeong	1	27.7	69	Yes	201208	10	0	0.00	
9	50	High	TMR	Gapyeong	1	27.8	63.8	Yes	201208	10	0	0.00	
10	70	Low	TMR	Cheongpyeong	2	27.8	63.8	Yes	201208	10	0	0.00	
						21.4	78.6	Yes	201309	4	1	25.00	0909-9 (G, O157)
11	30	Mid	TMR	Gapyeong	2	21.8	94.3	Yes	201209	9	1	11.11	0904-9 (G, O84)
						21.4	78.6	Yes	201309	7	5	71.43	0909-11 (D, O157), 0909-14 (D, O157), 0909-15 (D, O157), 0909-16 (D, O157), 0909-17 (G, O157)
12	50	Low	TMR	Gapyeong	2	21.8	94.3	Yes	201209	10	1	10.00	0904-16 (D, O185)
						20	98.9	Yes	201309	9	2	22.22	0911-3 (D, O169), 0911-11 (D, O157)
13	80	High	TMR	Gapyeong	2	21.8	76.9	Yes	201209	11	1	9.09	0905-7 (D, O157)
						20	98.9	Yes	201309	8	0	0.00	
14	60	High	TMR	Gapyeong	1	21.8	76.9	Yes	201209	12	0	0.00	
15	150	High	Hay	Yangpyeong	9	30.5	62.9	No	201208	24	3	12.50	0806-3 (D, O157), 0806-5 (D, O157), 0806-20 (G, O157)
						11.0	63.6	No	201210	28	1	3.57	1015-16 (G, O84)
						−4.2	75	No	201301	32	0	0.00	
						19.7	81.3	Yes	201305	36	8	22.22	0527-1 (D,O108), 0527-4 (D,O108), 0527-8 (D,O119), 0527-15-1 (D, O108), 0527-15-2 (D,O119), 0527-19 (D,O108), 0527-23 (D,O108), 0527-24 (D,O119)
						20.7	92.1	Yes	201408	34	13	38.24	0814-4 (D, O157), 0814-5 (D, O157), 0814-7 (D, O157), 0814-8 (D, O157), 0814-11 (B, O157), 0814-13 (D, O15), 0814-16 (D, O8), 0814-20 (D, O157), 0814-22 (D, O157), 0814-25 (D, O15), 0814-31 (D, O157), 0814-32 (D, O55), 0814-34 (D, O157)
						16.8	54.6	No	201410	40	4	10.00	1013-6 (C, O111), 1013-12 (B, O8), 1013-19 (G, O157), 1013-21 (D, O84)
						0.1	90.8	No	201412	27	3	11.11	1215-7 (D, O15), 1215-8 (D, O109), 1215-24 (B, O109)
						25.4	65.6	Yes	201507	40	5	12.50	0709-6 (D, O8), 0709-7 (D, O8), 0709-29 (B, NT), 0709-32 (C, O111), 0709-35 (C, O84)
						19.2	60.1	No	201509	33	0	0.00	

^a^ Hygiene level of sawdust: Low; very wet, dirty, and slippery, Mid; normal, High; very dry

^b^ Prevalence of STEC were significantly higher at the temperature above 20 °C and when rained within 3 days prior to sampling date (*p* < 0.001, Chi squared test, two tailed)

^c^ Temperature: average temperature of sampling day, rain: raining within 3 days prior to sampling date considered yes

^d^ TMR: total mixed ration

## Results

### STEC prevalence in cattle farms

Of 496 samples collected from 29 visits to 15 farms, 63 STEC were isolated from 17 visits to seven farms (Table 2). Most STEC were isolated from feces (54 from adult cow and three from calves), and six STEC were isolated from ground soil. No STEC were isolated from feed, water, or milk samples. The farm prevalence of STEC varied from 0.0 to 90.0%, but no farm management factors were found to be associated with STEC prevalence. STEC prevalence was significantly higher when the average temperature was above 20 °C (*p* < 0.001, two tailed Chi squared test, odds ratio [OR] = 2.3), and when rain was reported within 3 days prior to sampling (*p* < 0.001, two tailed Chi squared test, OR = 3.5). Moreover, while the STEC prevalence was higher in calves (3/19; 15.8%) than in adults (54/405; 13.3%), the difference was not significant. By breed, STEC prevalence was significantly higher in beef cattle (4/9; 44.4%) than in dairy cattle (50/396; 12.63%; *p* < 0.05, two-tailed Fisher’s exact test, OR = 5.5).

### Serogroup

Of the 63 STEC isolates, 61 were serogrouped. Thirty-five (55.6%) and 26 (41.3%) isolates were found to be STEC O157 and non-O157, respectively, whereas two isolates were non-typeable (NT). Among STEC non-O157, the most common serogroup was O108 (five isolates, 7.5%), followed by O8 and O84 (four isolates each, 6.0%), O15 and O119 (three isolates each, 4.5%), O109 and O111 (two isolates each, 3.0%), and O55, O169, and O185 (one isolate each, 1.5%).

### Prevalence of Shiga toxin and its variant genes

The Shiga toxin gene types of the 63 STEC isolates were determined by PCR. STEC harboring both *stx1* and *stx2* (28/63, 41.8%) were the most common strains, followed by STEC with either s*tx1* (13/63, 19.4%) or *stx2* (22/63, 32.8%; Table 3). STEC harboring *stx1* were only detected in STEC non-O157 (O55, O84, O108, and O111), while STEC O157 harbored *stx2* only or both *stx1* and *stx2* with a similar distribution. Of the 35 STEC harboring *stx1*, 34 (97.1%) carried the *stx1c* variant, while no *stx1d* variants were detected. All STEC harboring *stx2* (n = 50) carried at least one *stx2* variant, *stx2a* (45; 90.0%)*, stx2c* (34; 68.0%), and *stx2* *g* (5; 10.0%). *Stx2d, stx2e*, and *stx2f* were not detected. When the distribution of the *stx* variants was analyzed based on serotypes, *stx2a* was observed in O8, O119, O157, O169, and O185; *stx2c* in O157 (33/35, 94.3%), and O185 (1/1, 100.0%); and *stx2* *g* in O15 (3/3, 100.0%) and O109 (2/2, 100.0%).

**Table 3:** Shiga toxin genotypes of STEC isolates

Shiga toxin gene type	Serotype	No. of isolates	*stx* variants			
			*stx1c*	*stx2a*	*stx2c*	*stx2g*
*stx1* only	O55	1	1	–	–	–
	O84	4	4	–	–	–
	O108	5	5	–	–	–
	O111	2	2	–	–	–
	NT	1	1	–	–	–
	Subtotal	13	13	–	–	–
*stx2* only	O15	3	–	0	0	3
	O109	2	–	0	0	2
	O119	3	–	3	0	0
	O157	18	–	18	18	0
	O169	1	–	1	0	0
	O185	1	–	1	1	0
	Subtotal	28	–	23	19	5
Both *stx1* and *stx2*	O8	4	3	4	0	0
	O157	17	17	17	15	0
	NT	1	1	1	0	0
	Subtotal	22	21	22	15	0
Total		63	34	45	34	5

*NT* non-typeable for STEC serogroup

### Antimicrobial resistance of the STEC isolates

Disc diffusion tests were conducted using 14 antimicrobial agents. While resistance was observed for TE (five isolates, 17.9%), AMP (four isolates, 14.3%), and CTX (one isolate, 3.6%) in 28 STEC non-O157 strains, resistance was not observed in 35 STEC O157 strains. Four and five STEC O157 and non-O157, respectively, showed intermediate resistance to CTX. The STEC isolates showing complete or intermediate resistance to CTX were further tested for ESBL-producing STEC, but none of them had the ESBL phenotype (Table 4).

**Table 4:** Phenotypic and genotypic antimicrobial resistance profiles of STEC isolates

Antimicrobial class	Phenotypic profiles			Genotypic profiles		
	Antimicrobial^a^	No. of STEC isolates^b^ (%)		Antimicrobial resistance gene^c^	No. of STEC isolates (%)	
		STEC O157	STEC non-O157^b^		STEC O157	STEC non-O157
Β-lactams	Ampicillin	0 (0.0)	4 (14.3)	*ampC*	35 (100.0)	28 (100.0)
	Cefotaxime^d^	0 (0.0)	1 (3.6)	*Bla*_*TEM*_	0 (0.0)	11 (36.3)
Tetracycline	Tetracycline	0 (0.0)	5 (17.9)	*tetB*	0 (0.0)	4 (14.3)
				*tetC*	0 (0.0)	3 (10.7)
				*tetE*	34 (97.1)	0 (0.0)

^a^ All STEC showed susceptibility on chloramphenicol, imipenem, amikacin, amoxicillin–clavulanic acid, ceftazidime, gentamicin, nalidixic acid, trimethoprim–sulfamethoxazole, ceftriaxone, aztreonam, and cefpodoxime on standard disk-diffusion test

^b^ Antimicrobial resistance profiles were tested for 35 and 28 STEC O157 and non-O157 strains, respectively

^c^ Antimicrobial resistance genes of *tetA, tetD, tetG, cat, cml, bla*_*OXA*_ *, bla*_*CMY*_, and *qnr* were not detected from all STEC isolates

^d^ Four STEC O157 and five non-O157 showed intermediate resistance to cefotaxime

All of the 63 STEC isolates carried more than one antimicrobial resistance genes. Of 13 tested antimicrobial resistance genes, genes *tetA, tetD, tetG, cat, cml, bla*_*OXA*_ *, bla*_*CMY*_, and *qnr* were not detected. All STEC isolates carried *ampC* gene (63/63; 100.0%), and 11 isolates carried *bla*_*TEM*_, (11/63; 17.5%) gene. Of tetracycline resistance gene, *tetE* (34/63; 54.0%) was the most prevalent, followed by *tetB* (4/63; 6.3%) and *tetC* (3/63; 4.8%) (Table 4).

### Virulence gene profiles

The prevalence of virulence genes was investigated. The prevalence of each virulence gene was as follows: *eae* (77.8%), *tir* (76.2%), *espB* (74.6%), *espD* (65.1%), *ehxA* (92.1%), *katP* (55.6%), *espP* (84.1%), *iha* (66.7%), *subA* (7.9%), *stcE* (65.1%), and *saa* (9.5%).

**Fig. 1:**
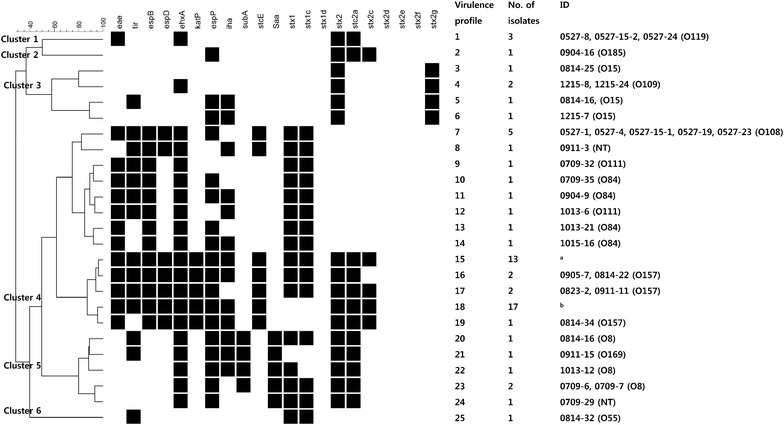
UPGMA dendrogram of STEC O157 and non-O157 based on the profiles of virulence genes. Six clusters were generated with the UPGMA method based on 50% similarity. ^a^0806-3, 0806-5, 0806-20, 0823-4, 0823-5, 0823-8, 0909-5, 0909-9, 0909-11, 0909-14, 0909-15, 0909-16, 0909-17 (O157). ^b^0827-1, 0827-2, 0827-3, 0827-5, 0827-6, 0827-7, 0827-8, 0827-9, 0827-10, 0814-4, 0814-5, 0814-7, 0814-8, 0814-11, 0814-20, 0814-31, 1013-19 (O157)

When clustering analysis was conducted based on the profiles of the virulence genes, including Shiga toxin and its variant genes, six clusters were generated (based on 50% similarity; Fig. 1). STEC strains in clusters 1, 2, and 6 were composed of a single serotype each, O119, O185, and O55, respectively. The strains showed 100% prevalence of *eae*, *ehxA, stx2,* and *stx2c* in cluster 1; *espP, stx2, stx2a*, and *stx2c* in Cluster 2; and *tir*, *stx1*, and *stx1c* in cluster 3. Strains in cluster 3 were characterized as having *stx2* *g* variants, and belonged to the O15 and O109 serogroups. Cluster 4 was composed of STEC O84, O108, O111, and O157 strains with a high prevalence of *eae*, *tir*, *espB*, *espD*, *ehxA, katP, espP, iha, stcE, stx2, stx2a,* and *stx2c*. Strains in cluster 5 were characterized as having *subA* and *saa* (Table 5).

**Table 5:** Prevalence of virulence genes in each cluster

Cluster	No. of isolates	Serotype (no. of isolates in each serotype)	Prevalence of virulence genes (%)										
			*eae*	*tir*	*esp* *B*	*esp* *D*	*ehx* *A*	*kat* *P*	*esp* *P*	*iha*	*sub* *A*	*stc* *E*	*Saa*
1	3	O119 (3)	100.0	0.0	0.0	0.0	100.0	0.0	0.0	0.0	0.0	0.0	0.0
2	1	O185 (1)	0.0	0.0	0.0	0.0	0.0	0.0	100.0	0.0	0.0	0.0	0.0
3	5	O15 (3), O109 (2)	0.0	20.0	0.0	0.0	40.0	0.0	40.0	40.0	0.0	0.0	0.0
4	47	O84 (4), O108 (5), O111 (2), O157 (35), NT (1)	97.9	93.6	100.0	87.2	100.0	74.5	93.6	78.7	0.0	87.2	0.0
5	6	O8 (4), O169 (1), NT (1)	0.0	33.3	0.0	0.0	100.0	0.0	100.0	50.0	83.3	0.0	100.0
6	1	O55 (1)	0.0	100.0	0.0	0.0	0.0	0.0	0.0	0.0	0.0	0.0	0.0

*NT* non-typeable

### Genetic relatedness of the STEC isolates

**Fig. 2:**
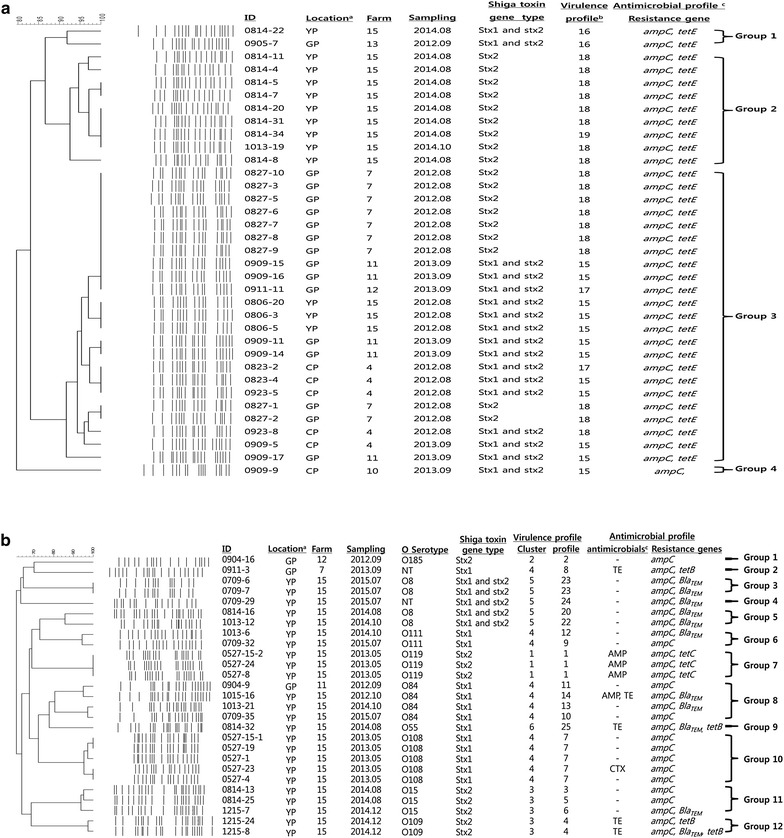
Phylogenetic analysis of STEC isolates from cattle. The UPGMA method was used with a 1.5% optimization and 1.5% tolerance window using Bionumerics software. ^a^ *CP* Cheongpyeong, *GP* Gapyeong, *YP* Yangpyeong. ^b^All STEC O157 strains were belonged to cluster 4. ^c^ *AMP* ampicillin, *CTX* cefotaxime, *TE* tetracycline. **a** STEC O157 strains were clustered into four groups (based on 90% similarity). **b** STEC non-O157 strains clustered into 12 groups (based on 80% similarity), STEC strains with the same serotype clustered together, except for STEC O8. The STEC O169 strains were not typeable by PFGE

The PFGE patterns of *Xba*I-digested STEC O157 and non-O157 were analyzed to determine how the clonal relatedness of STEC isolates changed with temporo-spatial variation. The DNA fingerprints of 35 STEC O157 strains showed high similarity, generating 16 different PFGE profiles with 15–20 discernible fragments (mean: 17.4, median: 17). The 16 PFGE profiles clustered into four groups, based on 90% similarity; each group was composed of one to 23 isolates (Fig. 2a).

Pulsed-field gel electrophoresis was performed for STEC strains belonging to 10 different serogroups, other than O157 and two strains that were non-typeable for the O serotype. Diverse PFGE patterns of *Xba*I-digested STEC non-O157 were observed, resulting in 20 different PFGE profiles with 14–23 discernible fragments (mean: 18.2, median: 19), except for one isolate (0911-15), which was untypeable by PFGE. A UPGMA dendrogram, generated using Dice coefficient analysis, clustered STEC non-O157 into 12 different groups, based on 80% similarity. Each group was composed of one to five strains, and STEC strains belonging to the same serotype were grouped together regardless of differences in the sampling period or geographical location of each farm (Fig. 2b).

## Discussion

The prevalence of STEC in 15 different cattle farms, virulence-gene profiles, antimicrobial resistance, and genetic relatedness of STEC isolates were analyzed to investigate the virulence potentials of STEC in cattle farm.

During the sampling period, 63 STEC were isolated from 469 cattle farm samples collected from 15 cattle farm in Gyeonggi province in South Korea. Numerous studies are ongoing to identify the factors associated with STEC prevalence. In this study, high temperature and rain were found to be associated with STEC prevalence. Similarly, a previous study reported higher STEC prevalence in hot seasons than in cold seasons [31–33]. In addition, rainfall has been considered an important transmission factor for STEC. The pathogens may be transported via sediments to vast geographical regions as far away as 32 km, resulting in an increased prevalence in the environment [34, 35]. Many published reports have shown that STEC O157 prevalence is higher in calves, especially in post-weaned calves, than in adult cattle [36–38]. However, no obvious link between age and STEC non-O157 prevalence has been reported, and some investigators even observed a higher prevalence of STEC non-O157 in adult groups [37, 39, 40]. In this study, the adult group showed a higher prevalence for STEC O157 (calves: 0/19, 0.0% vs. adults: 31/405, 7.7%) and a lower prevalence for STEC non-O157 (calves: 3/19, 15.8% vs. adult: 24/405, 5.9%). This discrepancy with respect to previous data may be due to the collection of a relatively small number of calf feces samples, compared to the number of samples collected from adults. Thus, further studies may be needed to investigate the effect of age on STEC non-O157 prevalence. Here, beef cattle showed higher STEC prevalence than dairy cattle. Although only a few beef cattle were included in this study, the results are consistent with data from previous studies [31, 33].

While many studies have focused on the O157 serotype, the significance of STEC non-O157 in human infection has become clear recently [3, 7]. In this study, 11 different serotypes of STEC were identified and >40% of the STEC were non-O157, highlighting the need for active surveillance of STEC non-O157 and understanding their virulence potential in humans. Of the identified serotypes, O8, O15, O55, O84, O109, O111, O119, and O157 have been reported frequently in dairy cattle worldwide [39]. Among them, several serogroups have also been reported frequently in human clinical cases. The O111 serogroup is the second most common serogroup in human infections, and is the most common cause of HUS. Moreover, it accounts for half the STEC non-O157 outbreaks. The O15, O84, and O119 serogroups also frequently cause human illness [7, 39]. In addition, human-pathogenic STEC O8, O15, and O109 serotypes have been detected in food samples, highlighting the possible transmission of STEC via the food chain [41].

The genetic variation of Shiga toxin causes changes in its amino acid composition, which may directly influence the virulence of STEC, resulting in a change in the toxin receptor tropism or toxicity of Shiga toxin [11]. In this study, high prevalence of *stx1c, stx2a*, and *stx2c* was detected. The *stx1c* variants are associated with ovine-originated STEC strains [42–44], but the high prevalence of *stx1c* in buffaloes, cattle, and goats was reported to account for 80% of the *stx1* variants, indicating a wide distribution of *stx1c* variants in STEC of bovine origin [45]. *stx1c* variants have been found as *stx1c* only or in combination with *stx1, stx2*, or *stx2d.* However, in this study, combinations involving *stx1, stx1c, stx2, stx2a,* and *stx2c* (16 isolates); *stx1, stx1c, stx2*, and *stx2a* (six isolates); and *stx1* and *stx1c* (13 isolates) were newly found. In addition, *stx1c*-producing STEC is considered a subset of *eae*-negative STEC, and is responsible for asymptomatic or mild disease [42, 44, 46]. However, in this study, 29 *stx1c*-producing STEC harbored *eae*. The *stx1c* variants in *eae*-positive STEC strains might be resulted from the dynamics of virulence genes. Of the *stx2* variants*, stx2a, stx2c,* and *stx2d* variants have been implicated in high STEC virulence [21, 47]. While *stx2d* was not detected in the current study, the high prevalence of *stx2a,* and *stx2c* suggested the wide distribution of potentially pathogenic STEC strains in cattle farms. The *stx2g* variant was detected from five STEC non-O157 (three O15 and two O109 STEC). Previously, the *stx2g* variant has been identified from various sources, including cattle, beef or beef-containing products, and humans, suggesting a possible route of exposure of these STEC types via the food chain [41, 48].

To evaluate the virulence potentials of STEC strains isolated from cattle farms, the phenotypic and genotypic antimicrobial resistance features and the prevalence of virulence genes were investigated. In this study, all STEC isolates were susceptible to all tested antimicrobials, except for AMP, TE, and CTX. Resistance to AMP and TE in diverse sources, including cattle or beef products, have commonly been reported in previous studies [28, 29, 49], but resistance to CTX is uncommon, with only one isolate (of 722) from a bovine source being reported to date [50]. CTX, a third generation cephalosporin, is used as an indicator to identify ESBL production. Although ESBL production was not identified in this study, the presence of CTX-resistant STEC indicates the need for implementing antimicrobial resistance control strategies to prevent the generation and spread of ESBL-producing STEC.

In addition, all the STEC strains that exhibited resistance to AMP, TE, and CTX were STEC non-O157 strains. Genotypic antimicrobial features also varied by its serotype. While antimicrobial resistance genes of *tetB*, *tetC*, and *bla*_*TEM*_ were only observed in STEC non-O157 strains, *tetE* was detected only in STEC O157 (34/35; 97.1%). These results suggest that antimicrobial resistance is higher in STEC non-O157 than in STEC O157, consistent with previous studies [49, 50]. While the antimicrobial resistance gene *ampC* was amplified from all tested STEC isolates, only four STEC non-O157 strains exhibited phenotypic resistance. Since many genes and mechanisms, including efflux pumps or intrinsic resistance, are involved in the development of resistance features, genetic determinants may not represent the phenotypic resistance features [51, 52].

The prevalence of virulence genes in each serotype was either 0 or 100%, except for *tir, espP*, and *iha*, indicating the sero-specific feature of virulence genes. To estimate the virulence potentials of STEC strains that might cause a risk to public health, clustering analysis was performed based on the virulence gene profiles. Six clusters were generated, and sero-specific features were observed in each cluster. Cluster 1 was composed of O119 STEC, which has 100% prevalence of the well-known virulence factors *eae* and *ehxA*. The association between intimin (encoded by *eae*) and STEC virulence has been reported previously, and serogroup O119 has been detected in human infections [4, 7]. This indicates that the STEC isolates in Cluster 1 might have the potential to cause human illness. Most of the other STEC strains were grouped in Cluster 4 (47/63, 74.6%), and these strains harbored most of the virulence genes at a high frequency, except for *subA* and *saa*. The *katP* and *stcE* gene products are believed to promote STEC virulence by assisting STEC colonization in the intestines and degrading the protective layers in the intestines, respectively [27, 53]. A high prevalence of these two genes was reported for sero-pathogroups A and B, which are responsible for severe STEC illness [54]. In this study, all of the O157 and O111 serotypes, which belonged to sero-pathotypes A and B, also belonged to Cluster 4, indicating the high virulence potential of the STEC in Cluster 4. Cluster 5 was characterized by the presence of *subA* and *saa*, and consisted of O8, O169, and NT STEC. *subA* is purported to increase STEC virulence. Saa also increases STEC virulence by assisting in adherence to host cells in *eae*-negative STEC [55, 56]. On the other hand, the STEC in Clusters 2, 3, and 6 appeared to be less pathogenic to humans. High prevalence of *espP, iha*, and *ehxA* was reported regardless of sero-pathotype, suggesting the absence of a strong association between these genes and STEC virulence [54].

Pulsed-field gel electrophoresis analysis was performed to understand the clonal relatedness of STEC strains isolated from cattle farms located in different regions of the Gyeonggi province in Korea during 2012–2015. For the STEC O157 strains, those isolated from the same farm during the same sampling period had indistinguishable PFGE profiles except for a few isolates from farms 4, 7, 11, and 15, which showed one to three different bands. Considering that a single nucleotide mutation at a restriction enzyme site causes three fragment differences [57], a minor genetic variation may have occurred within the farm. In addition, STEC O157 from farm 4 showed high similarity between the 2012 and 2013 isolates. Phylogenetic analysis combined most isolates into group 3, which consisted of isolates from five farms in three different geographical locations. These results indicated the possible presence of a prototype of STEC O157 in the Gyeonggi province with a minor genetic variation, which led to within- and between-farm transmission during 2012–2013. However, STEC O157 isolates from farm 15 showed a higher degree of polymorphism; these isolates clustered in groups 1 and 2 (STEC strains isolated in 2014) and group 3 (STEC strains isolated in 2012). These results indicated that the prototype of STEC O157 in farm 15 might have changed in 2014. Because all the farms were located in the Gyeonggi province and the longest distance between farms was approximately 60 km, temporal effects may have been less important. While a high degree of genetic diversity was observed in STEC non-O157, they were grouped together for strains with the same serotype. STEC O8, O15, O84, and O111 were isolated multiple times and shared genotypic similarity over the 3-year period within the serogroup, implying that these STEC strains have endured and continue to survive, causing within-farm transmission.

## Conclusions

Shiga-toxin-producing *Escherichia coli* prevalence differed greatly between farms, and temperature and rainfall affected the farm prevalence. A considerable number of STEC non-O157 stains were isolated, and different virulence and antimicrobial resistance features were observed between STEC O157 and non-O157 strains. While a high prevalence of virulence genes was observed in STEC O157 strains, the antimicrobial resistance rate was higher in STEC non-O157 strains. In addition, the *stx1c* variant was detected in *eae*-positive STEC, suggesting genetic dynamics among virulence genes in STEC isolates. Finally, PFGE analysis revealed the presence of a prototype STEC, which continues to evolve by genetic mutation and causes within- and between-farm transmission within the Gyeonggi province. Our results suggested that STEC from cattle have a high virulence potential and represent a threat to public health. Therefore, continual surveillance of both STEC O157 and non-O157 would be beneficial for controlling and preventing STEC illness.

## Abbreviations

AM: ampicillin
AMC: amoxicillin–clavulanic acid
AN: amikacin
ATM: aztreonam
C: chloramphenicol
CAZ: ceftazidime
CPD: cefpodoxime
CRO: ceftriaxone
CTX: cefotaxime
ESBL: extended-spectrum beta-lactamase
GM: gentamicin
IMP: imipenem
NA: nalidixic acid
PCR: polymerase chain reaction
PFGE: pulsed-field gel electrophoresis
STEC: Shiga toxin-producing *Escherichia coli*
STX: trimethoprim–sulfamethoxazole
TE: tetracycline
UPGMA: unweighted pair group method with arithmetic mean analysis
